# Regulatory T-cell phenotypes in prenatal psychological distress

**DOI:** 10.1016/j.bbi.2023.11.033

**Published:** 2023-11-26

**Authors:** Kyle S. Wiley, Dayoon Kwon, Delaney A. Knorr, Molly M. Fox

**Affiliations:** a Department of Anthropology, University of California, Los Angeles, United States; b Department of Psychiatry and Biobehavioral Sciences, University of California, Los Angeles, United States; c Department of Epidemiology, UCLA Fielding School of Public Health, University of California, Los Angeles, United States

**Keywords:** Anxiety, Pregnancy, Regulatory T-cells, Prenatal mood, Stress, Depression

## Abstract

**Background:**

Experiencing symptoms of psychological distress during pregnancy is common and has been linked to dysregulated immune functioning. In this context, immunoregulatory function is especially relevant because of its crucial role in establishment and maintenance of healthy pregnancy. However, little research has examined associations between women’s prenatal psychological distress and immunoregulatory biomarkers. We investigated how symptoms of depression, anxiety, and stress relate to circulating levels of regulatory T-cells (Tregs).

**Materials and methods:**

Pregnant Latina women were assessed at around 12 weeks of pregnancy (N = 82). These assessments included blood draws and self-report questionnaires assessing symptoms of depression, state anxiety, pregnancy-related anxiety, and perceived stress. Flow cytometry on PBMCs was used to quantify circulating Tregs, defined as CD3^+^CD4^+^CD25^hi^CD127^lo^FoxP3^+^, and subpopulations positive for one of the following intra- or extracellular markers, CD45RA, CTLA-4, Helios, PD-1, TIM-3, and TIGIT. We collected 82 samples at 12 weeks. Multivariable linear regressions tested for associations between symptoms of psychological distress and Treg concentrations, adjusted for gestational age.

**Results:**

State anxiety symptoms at 12 weeks were negatively associated with parent Treg cell levels (b = −4.02, p = 0.023) and subpopulations Helios^+^ (b = −3.29, *p* = 0.019) and TIM3^+^ (b = −3.17, *p* = 0.008). Perceived stress was negatively associated with the PD-1^+^ subpopulation at 12 weeks (b = −4.02, *p* = 0.023). Depression was not related to Tregs or the subpopulations.

**Conclusion:**

Our observation that symptoms of anxiety and stress are related to tolerogenic immunology suggests a possible biomechanism explaining correlations of maternal mood disorders with adverse outcomes for mothers and offspring.

## 1. Introduction

Depression, anxiety, and stress are common in the perinatal period (Accortt and Wong, 2017; Reck et al., 2008) and pregnancy is a vulnerable period for the onset of mood disorder symptoms (Fishell, 2010). Roughly 15 % of women experience depression (Okagbue et al., 2019) and 16 to 25 % meet diagnostic criteria for an anxiety disorder during pregnancy (Fawcett et al., 2019), although rates are likely as much as two times higher for vulnerable populations (Howard et al., 2014). Importantly, as many as 50–70 % of cases are estimated to be undiagnosed and several social and cultural barreiers limit women’s access to treatment (Cox et al., 2016). Prenatal depression and anxiety are particularly understudied among minoritized communities, including Latinas, the demographic focus of this study. In the prenatal period, prevalence rates of depression range from 12 to 59 %, which is substantially higher than the prevalence of 10–15 % reported general population (Blackmore and Chaudron, 2014). Previous studies have shown that Latinas also report higher levels of anxiety and pregnancy-related anxiety than their white counterparts (Grobman et al., 2016; Ramos et al., 2019). There is also a lack of culturally tailored interventions for Latinas experiencing prenatal depression and anxiety symptoms (Ponting et al., 2022).

Experiencing a mood disorder during pregnancy is also associated with an increased risk for postnatal depression and anxiety (Heron et al., 2004). Furthermore, prenatal depression and anxiety are associated with an increased risk of pregnancy complications and adverse birth outcomes (Accortt et al., 2021; Cole-Lewis et al., 2014; Grigoriadis et al., 2018; Ramos et al., 2019), as well as risk of developmental and health problems for offspring (Dunkel Schetter and Tanner, 2012; Gluckman et al., 2005). The immune system is hypothesized to be one mechanism that may link perinatal mood disorders and adverse outcomes for both the mother and offspring (Christian, 2012, 2020). However, research on the immune correlates of perinatal mood disorders remains relatively limited.

Pregnancy is a unique life stage in which the maternal immune system must tolerate a semi-allogenic fetus that has antigens of paternal origin. In this period, the maternal immune system must accomplish the seemingly paradoxical task of defending both the mother and fetus from pathogens without rejecting the semi-allogenic fetus (La Rocca et al., 2014). This is accomplished through the induction of a tolerogenic environment created by regulatory T-cells (Tregs). Tregs are CD4^+^ T-cells characterized by high expression of CD25 and Forkhead Box P3 (FoxP3) along with low expression of CD127 and usually constitute about 5–15 % of CD4^+^ T-cells in the periphery (Sakaguchi, 2004; Wing and Sakaguchi, 2010). Treg populations significantly expand during normal pregnancy in both the decidua and periphery and peak in the second trimester (Sasaki et al., 2004; Somerset et al., 2004). Longitudinal studies suggest that Treg populations may continue to expand postpartum (Lima et al., 2017; Wegienka et al., 2011). Low concentrations of Tregs are associated with adverse pregnancy and birth outcomes, highlighting the costs of dysregulated immunosuppressive function during pregnancy (Green et al., 2021). We argue that to fully understand the relationship between inflammation and mental health in pregnancy, it is necessary to examine the immune system’s immunosuppressive arm, given its critical role in the establishment and success of pregnancy. In this analysis, we investigated associations between maternal peripheral Treg populations and psychological distress at two points in pregnancy.

Subpopulations of Foxp3^+^ Tregs can be characterized by various intra- and extracellular proteins that modify their immunosuppressive function. These include markers such as Protein tyrosine phosphatase, receptor type, C (CD45RA), cytotoxic T-lymphocyte associated protein 4 (CTLA-4), T cell immunoreceptor with Ig and ITIM domains (TIGIT), T cell immunoglobulin and mucin-domain containing-3 (TIM-3), and Programmed cell death protein 1 (PD-1) (Miko et al., 2019; Xu et al., 2017; Zhang and Sun, 2020). While Tregs subpopulations characterized by these markers have been inversely associated with adverse pregnancy outcomes and complications in murine and human studies (Jasper et al., 2006; Wang et al., 2022; Zenclussen et al., 2005), potential associations with maternal psychological distress have not been investigated.

The links between the adaptive immune system and mood disorders are well-established outside of the context of pregnancy. Converging evidence in humans and animals supports the role of immune dysregulation in pathophysiology of depression (Miller and Raison, 2016; Slavich and Irwin, 2014) and higher levels of pro-inflammatory cytokines, such as IL-6, IL-1β, and TNF-α, T helper 17 (Th17) cells, and lower concentrations of Tregs have been implicated in this process (Beurel and Lowell, 2018; Dowlati et al., 2010; Grosse et al., 2016). Tregs are inversely correlated with the pro-inflammatory state of monocytes in patients with major depression (Grosse et al., 2016). Antidepressants also help maintain the balance between Tregs and pro-inflammatory cells in animal models of depression (Zhang et al., 2013). These studies suggest that regulation of immunosuppressive function via Tregs may play a causal role in the etiology of depression.

The associations between anxiety and the adaptive immune system are less well-studied than the links with depression. Some studies have shown that individuals with anxiety disorders also have higher levels of circulating IL-6 or higher levels of IL-6 gene expression (Murphy et al., 2015; O’Donovan et al., 2010). However, authors of a recent population-based study were unable to replicate associations between high levels of IL-6 and anxiety disorders (Lee, 2020). There is a dearth of studies investigating other arms of the adaptive immune system. However, some evidence suggests that anxiety patients have higher levels of circulating Th17 cells (Vieira et al., 2010). There are, to our knowledge, no studies on the association between circulating Treg levels and anxiety in non-pregnant human individuals. However, rodent studies suggest that Treg-depleted mice display increased anxiety in response to stressors (Kim et al., 2012a).

There is relatively little research on the link between women’s perinatal psychological distress and the adaptive immune system during pregnancy or postpartum. This is despite the importance of the mother’s immune system in maintaining a healthy pregnancy and the abundance of evidence suggesting its role in the etiology of mood disorders outside of the context of pregnancy (Osborne et al., 2019). One study examined methylation of the FoxP3 gene across a Treg-cell-specific demethylated region, whose expression in an unmethylated form ensures that naïve T cells develop into Tregs, and found that prenatal racial and ethnic discrimination exposure predicted postnatal mood disorders only in individuals with higher than average levels of FoxP3 methylation (Sluiter et al., 2020). Another study found that women with postpartum depression did not exhibit the characteristic rebound in Tregs that those without postpartum depression exhibit (Osborne et al., 2020). In a longitudinal study of adaptive immune cells from the second trimester of pregnancy to the postpartum, Sherer et al. (2022) reported that women with anxiety displayed a decrease in the ratio of B to T-cells from pregnancy to postpartum as well as a lower ratio of Th17 to Treg cells in the postpartum. Together, these studies suggest that Tregs could be implicated in perinatal mood dysregulation. No studies to our knowledge have examined associations between Treg subpopulation frequencies and mood disorders or psychological distress in pregnancy.

In this study, we expand on the current literature to examine how Treg subpopulations are associated with symptoms of multiple forms of perinatal psychological distress, including symptoms of depression, state anxiety, pregnancy-related anxiety, and perceived stress, around 12 weeks’. We hypothesize that psychological distress would be broadly inversely associated with Tregs. We investigated associations between FoxP3^+^ Tregs and Treg subpopulations and symptoms of depression, state anxiety, pregnancy-related anxiety, and perceived stress in a cohort of pregnant women living in the greater Los Angeles area. Six FoxP3^+^ Treg subpopulations were defined as Tregs that were also positive for one of the following intra- or extracellular markers: Helios, CD45RA, CTLA-4, PD-1, TIGIT, and TIM-3.

## 2. Methods and materials

### 2.1. Cohort

Data for this project derive from Wave 2 of the Mothers’ Cultural Experiences (MCE) study, an NIH-funded cohort study of Latina pregnant women in Southern California whose overarching goal is to examine the links between socio-cultural and environmental stressors with maternal-fetal and postnatal health and development. MCE involved two waves. Wave 1 was a cross-sectional study of pregnant and postpartum women. Wave 2 was a prospective, longitudinal study that followed women recruited during early pregnancy through 18-months postpartum. Eligibility for MCE Wave 2 included women who were age 18 years or older, English or Spanish-speaking, and self-identified as Latina, Hispanic, Chicana, or Mexican. Ethnicity eligibility was restricted due to study goals unrelated to the current project. MCE Wave 2 participants were recruited in prenatal clinic waiting rooms, gave informed written consent, and received modest compensation. Human subjects ethics approval was received from the institutional review boards of participating institutions (UCLA Medical IRB 3: IRB#18–000434; Olive View-UCLA Education & Research Institute IRB: Project 1086735–2; the other study sites had reliance agreements with UCLA). All protocols comply with the tenets of the Declaration of Helsinki. From an original longitudinal cohort of 107 pregnant women, our final analytic cohort included 82 pregnant women at 12 weeks. The 12 week assessments occurred from 15-Jan-2019 to 03-Mar-2020. As the 25-week follow-up included only 45 participants, after omitting women who were missing flow cytometry data, we elected to exclude it from this analysis. A high rate of attrition between the time points was due to the onset of the COVID-19 pandemic, and no in-person assessments occurred after the authors’ university’s temporary research shutdown began.

### 2.2. Protocol

This project uses data from the two prenatal assessments occurring around 12 weeks’ gestational age. Time point 1 gestational ages ranged from 5 to 18 weeks. At each prenatal assessment, participants completed a written questionnaire and gave specimens of saliva, urine, and peripheral blood. Demographic and health history data are self-reported, deriving from these questionnaires. All study materials were available in both English and Spanish, and study personnel were bilingual. The blood samples relevant to the current project were drawn by antecubital venipuncture into sodium heparinized vacutainers (Becton, Dickinson, and Company, Franklin Lakes, NJ) by hospital or clinic phlebotomists. The sodium heparin tubes were drawn first, before any other labs. These samples were kept at room temperature and transported immediately to the Cousins Center laboratory at University of California Los Angeles, arriving within a few hours of the blood draw. Peripheral blood mononuclear cells (PBMCs) were isolated following standard protocol, aliquoted, and stored at −80°C until analysis.

### 2.3. Maternal psychological measures

The mental health measures for this study (depression, state anxiety, pregnancy-related anxiety, and perceived stress) were all assessed using widely used and previously validated self-report, questionnaire-based instruments administered at both 12 weeks. For each scale, we determined reliability within our cohort by calculating Cronbach’s alpha (ɑ). For depression, we used the Edinburgh Perinatal Depression Scale (Cox et al., 1987; Garcia-Esteve et al., 2003; Murray and Cox, 1990), which calculates scores based on 9 items (ɑ = 0.86), omitting the self-harm question as previous studies have also done (Qiu et al., 2023). A sum is taken for each item, resulting in a range of 0 and 27 with a clinical risk cut-off score of depressive illness, or a high risk of developing a depressive disorder, as greater than 10. For anxiety, we used the Spielberger State-Trait Anxiety Inventory-Short Form (STAI) (Marteau and Bekker, 1992), a commonly used anxiety measure in pregnancy research (Meades and Ayers, 2011) shown to have strong psychometric value for both the English and Spanish language versions (Novy et al., 1995). The complete range of possible scores for the STAI is 1–4 and again, we found good reliability among our cohort (ɑ = 0.69). For the Pregnancy-Related Anxiety scale (PRA) (Rini et al., 1999; Wadhwa et al., 1993), each of the 10 items was scored from 1 to 4 and then averaged (ɑ = 0.83). Since its development, many other labs have validated it as a useful measure (Brunton et al., 2019), including the Spanish translation (Vázquez et al., 2018).

### 2.4. Staining and flow cytometry

To quantify T-cell populations, PBMCs were stained using antibodies from BioLegend, including CD3-Brilliant Violet 650, CD4-APC-Cyanine7, CD8-Alexa Fluor 700, CD25-Brilliant Violet 421, CD127-Brilliant Violet 605, CD45RA-Brilliant Violet 785, CTLA-4-APC CD152, FoxP3-PE, Helios-Alexa Fluor 488, PD-1-Brilliant Violet-711, Tim-3-PE/Dazzle CD366, and TIGIT-PE/CY7. Stained cells were then quantified by color flow cytometry using 4 laser AttuneNxT Accoustic Focusing cytometer (Invitrogen) using the AttuneNxT software. Cell populations were identified using the FlowJo software package (Tree Star, Ashland, OR, USA). The gating strategy for the cell populations of interest is provided in Supplemental Fig. 1. The positivity borderline was determined from fluorescence minus one (FMO) control tubes. Tregs are defined as CD3^+^CD4^+^CD25^hi^CD127^lo^FoxP3^+^ and subpopulations of Tregs are created under this parent Treg gate. Populations were quantified as the percentage of cells identified from the parent gate. Subpopulations were quantified as CD3^+^CD4^+^CD25^hi^CD127^lo^FoxP3^+^ that were also positive for the marker of interest.

### 2.5. Statistical methods

Highly skewed Treg variables (CD45RA, TIGIT, TIM3) were natural log transformed to approximate a normal distribution. All psychological measures/instruments and Treg variables were standardized to have a mean of 0 and SD of 1 to ease comparison across different mental health questionnaires. We used linear regression models to assess the association of mental health in women across pregnancy with their concurrent immune response. All models were adjusted for gestational age (weeks). We used R version 4.1.2 for analysis.

Covariates for sensitivity analyses were included based on their hypothesized role in exerting direct and independent effects on maternal immune function and psychological distress during pregnancy. For this reason, gestational age at sample collection, primiparity or multiparity, and the number of children were included as covariates. Treg levels have been shown to vary by gestational age and increase over pregnancy and nulliparous women have lower Treg cell percentages than parous women (Wegienka et al., 2011). As it is unknown if Treg levels increase with the number of subsequent pregnancies, we ran an additional model to account for the number of children.

## 3. Results

Sample demographic and clinical characteristics are presented in Table 1. A majority of our study participants were U.S.-born (45 %), graduated high school (63 %), employed (50 %), and single including never married, separated, divorced, and widowed (51 %). The average age of participants was 31 years (range: 21–42, SD = 5.68). For most of the participants, this was not their first pregnancy (76.8 %), and they had, on average, one child (range: 0–6, SD = 1.49). Approximately 23 % of women were classified as likely to have clinical depression, similar to other reports of high rates of depression in other perinatal Latina populations (Blackmore and Chaudron, 2014). We did not observe associations between mental health measures and CD3^+^, CD4^+^, or CD8^+^ T-cells in multivariable linear regression analyses adjusting for gestational age (p-values > 0.05, data not shown).

**Fig. 1.**
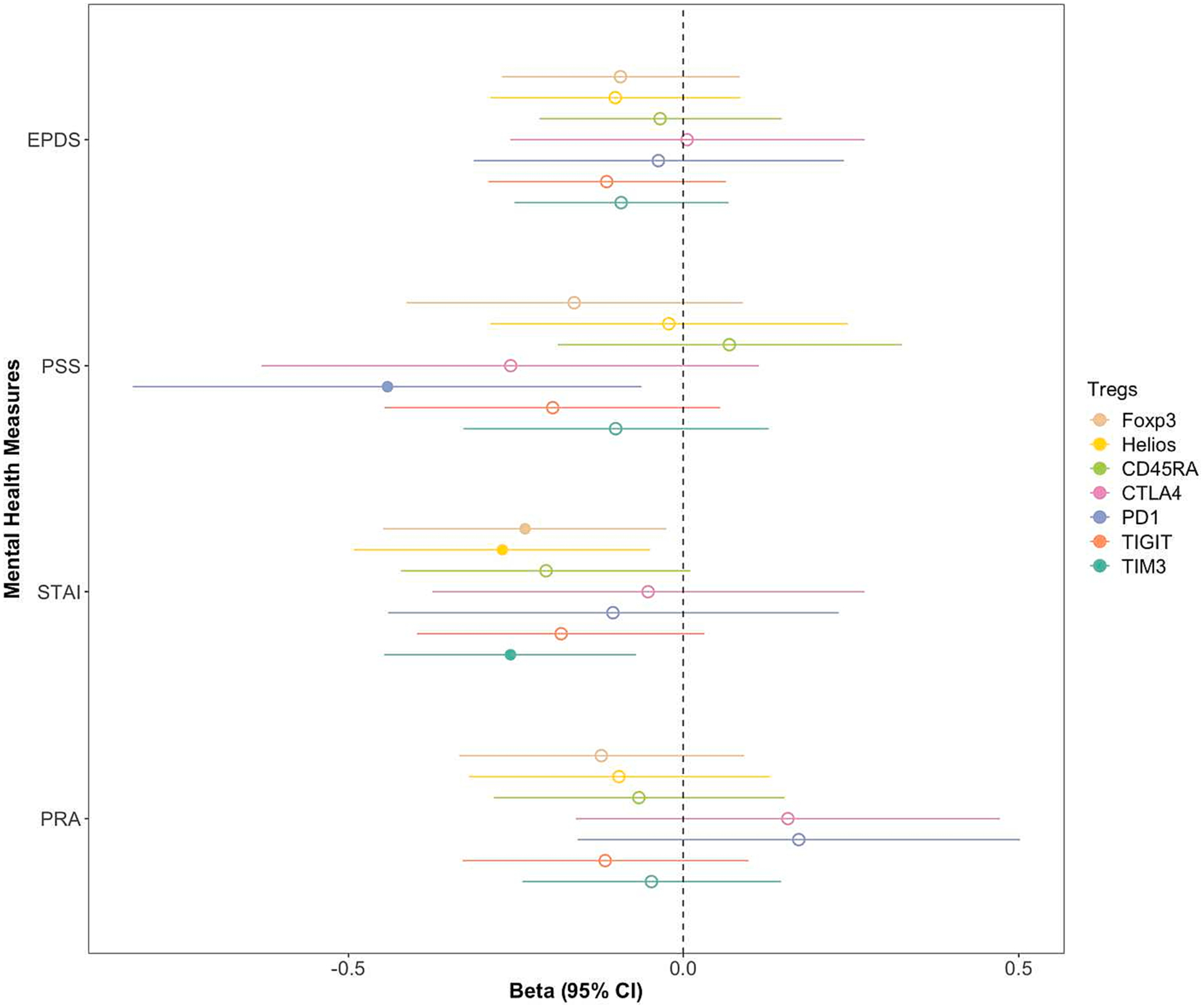
Association of different mental health measures in women at 12 weeks of pregnancy with their concurrent Tregs. All models adjusted for gestational age. EPDS = Edinburgh Postnatal Depression Scale, PSS = Shortened Perceived Stress Scale, STAI = Spielberger State-Trait Anxiety Inventory, PRA = Pregnancy-Related Anxiety Scale.

The results of the multivariable linear regression analyses examining how mental health measures relate to Treg levels, adjusting for gestational age, are shown in Fig. 1. Perceived stress was negatively related to the percentage of PD-1^+^ Treg cells (β = −4.02, *p* = 0.023) at 12 weeks. State anxiety was negatively associated with the percentage of FoxP3^+^ Tregs in the parent gate (β = −2.88, *p* = 0.031) as well as subpopulations of Helios^+^ (β = −3.29, *p* = 0.019) and TIM-3^+^ (β = −3.17, *p* = 0.008) Treg cells at 12 weeks. Additionally, sensitivity analyses using the number of children and parity as additional covariates (Table S2) did not change the pattern of associations between mental health conditions and immune architecture.

## 4. Discussion

This study examined associations between symptoms of maternal prenatal psychological distress and percentages of maternal circulating FoxP3^+^ Tregs and six Treg subpopulations expressing Helios, CD45RA, CTLA-4, PD-1, TIGIT, or TIM-3. Broadly, symptoms of prenatal psychological distress were negatively associated with concentrations of circulating Tregs. More specifically, we found that more feelings of perceived stress were associated with lower percentages of the PD-1^+^ Treg subpopulation and that symptoms of state anxiety were associated with lower percentages FoxP3^+^ Tregs as well as Helios^+^ and TIM-3^+^ subpopulations at 12 weeks.. Symptoms of depression were not associated with Tregs. The results of our study suggest that, most robustly, state anxiety symptoms in early pregnancy are associated with alterations to the immunosuppressive regulatory T-cell compartment. Perceived stress was associated with one Treg subtype at 12 of pregnancy.

Our results highlight the earlier part of pregnancy as the period in which maternal psychological distress is associated with maternal adaptive immune function. We identified significant associations between Treg subpopulations and maternal symptoms of psychological distress at 12 weeks of pregnancy. Previous studies have observed that Treg populations expand in the first trimester of pregnancy and pregnant individuals have higher levels of peripheral Tregs than non-pregnant individuals (Somerset et al., 2004). This initial expansion may be influenced by symptoms of psychological distress. However, as our study is cross-sectional, we are unable to determine whether distress symptoms are associated causally with reduced Tregs or vice versa. Future studies should follow individuals, their symptoms, and Treg levels from preconception through pregnancy to establish whether changes in Treg populations occur prior to or after the onset of mood disorder symptoms. Establishing the order of these events may potentially contribute to elucidating the causality of these relationships.

Our findings suggest that concurrent anxiety symptoms in early pregnancy may be associated with concentrations of circulating FoxP3^+^ Tregs and several Treg subpopulations, but not other populations of T-cells, such as cytotoxic or helper T-cells. The results of this study contribute to a small literature on associations between perinatal anxiety and immunoregulatory accrual in pregnancy, many of which have focused on how shifts in these cell populations predict postnatal symptoms of psychological distress. Only two studies have examined immune architecture in relation to anxiety in pregnancy and none have examined Treg subpopulations in early pregnancy. Sherer et al. (2022) assessed shifts in immune architecture from the second trimester to six weeks postpartum in anxious and non-anxious pregnant women and did not find significant differences in the Treg compartment during pregnancy or postpartum. Another recent study also did not observe differences in Th1 or Th2 cell concentrations between women with and without postpartum depression or anxiety (Min et al., 2022). Our results suggest that associations between anxiety and Tregs are detectable at 12 weeks of pregnancy.

Our results suggest that the mother’s Treg compartment relates most closely to her symptoms of stress, anxiety, and pregnancy-related anxiety, particularly for those that express markers that modify immunosuppressive capacity (PD-1, Helios, and Tim-3), in addition to the parent FoxP3 Treg population. Such intra- and extracellular proteins are known to be associated with the immunosuppressive function of Tregs and maintenance of pregnancy (Li et al., 2021; Zhang and Sun, 2020). We found that Helios^+^ and TIM-3^+^ Tregs were negatively associated with anxiety symptoms while perceived stress was negatively associated PD-1^+^ Tregs. No studies, to our knowledge, have examined associations between subpopulations of Tregs and depression, anxiety, or perceived stress. However, examination of Treg subpopulations may help explain the link between maternal psychological distress and suboptimal pregnancy and birth outcomes. For example, TIM-3 is known to enhance the suppressive function of Tregs and inhibit the proliferation and function of natural killer cells and effector T-cells (Teff) (Gautron et al., 2014; Ndhlovu et al., 2012; Zhu et al., 2005) and patients with recurrent pregnancy loss have lower levels of decidual TIM-3^+^ Tregs in early pregnancy (Hu et al., 2020). PD-1 signaling, meanwhile, suppresses the proliferation and function of T-cells, while also enhancing the suppressive capacity of Tregs (Francisco et al., 2010). In murine models, blocking the PD-1 pathway is associated with fetal loss and reduction in litter size, likely via excessive activation of Teff cells at the maternal-placental interface (D’Addio et al., 2011; Wafula et al., 2009). A recent study of human pregnancy has suggested that the PD-1/PD-L1 (the ligand of PD-1) pathway is positively associated with time-to-delivery (Espinosa et al., 2023). *In vitro* studies suggest that Helios^+^ Tregs also have increased stability and immunosuppressive capacity via the production of cytokines relative to Helios^−^ cells (Kim et al., 2015; Kim et al., 2012b; Zabransky et al., 2012). Lower levels of decidual Helios^+^ Tregs are associated with spontaneous miscarriage (Inada et al., 2015). Future studies of stress, mental health, and immune populations during pregnancy should consider subpopulations of Tregs.

We did not detect an association between depression and Treg concentrations at 12 weeks of pregnancy, which suggests that depressive symptoms may not be associated with inadequate immunosuppressive capacity, at least during pregnancy. This is somewhat in contrast to previous studies that have demonstrated increased inflammation related to depression during pregnancy and the early postpartum (Lahti-Pulkkinen et al., 2020; Leff Gelman et al., 2019), so we would have expected levels of immunosuppressive Tregs to be lower in relation to depressive symptoms. No previous studies have examined associations between concurrent depression symptoms and Treg populations in early or mid-pregnancy. However, two studies examined associations between third-trimester and postpartum Tregs and depression. One showed that women with postpartum depression had lower levels of naïve Tregs two months after birth relative to healthy postpartum controls (Osborne et al., 2020). Another found that women with postpartum depression two weeks after birth had higher levels of FoxP3^+^ Tregs relative to healthy controls during pregnancy and the postpartum (Krause et al., 2014). However, their study used different criteria for identifying Treg populations (CD4^+^CD15^+^CD127^−^) than ours. Collectively, this suggests that Tregs may not be related to depressive symptoms in early to mid-pregnancy, but may be more closely related to symptoms in the postpartum. However, such inferences are limited by the consistency of criteria used to define Treg populations. It is also possible that depression may influence the functional capacity, rather than production or absolute number of circulating Tregs. Future studies are needed to replicate these findings on Tregs and depression in early pregnancy, as well as to isolate Tregs and test their functional capacity *ex vivo*.

Alterations in Treg populations and their immunosuppressive capacity may play a role in the developmental origins of health and disease related to maternal psychological distress. Depression and anxiety have been associated with infant outcomes such as negative temperament and impaired stress regulation (Davis et al., 2007; Davis et al., 2004; Wiley et al., 2023). While the mechanisms underlying this process are unclear, they may involve higher levels of inflammation during pregnancy (Bolton and Bilbo, 2014; Parisi et al., 2021). Tregs may play an important role in this process by regulating the balance of pro-inflammatory activity during pregnancy. For example, the percentage of maternal, but not paternal, Tregs were correlated with concentrations of Tregs in newborns (Santner-Nanan et al., 2013), which may have implications for mental health later in life. Future studies are needed to investigate associations between the maternal Treg compartment and other infant outcomes.

This study has several limitations. It is important to note that we examined peripheral Treg and Treg subpopulations. It is largely unknown how peripheral Tregs may reflect decidual Tregs or exert influence at the maternal-fetal interface. There is some evidence to suggest that peripheral Tregs with fetal-specific antigens may be preferentially recruited to the maternal-fetal interface (Tilburgs et al., 2008). Future work should also investigate the functional capacity of the peripheral Treg populations identified in our study during pregnancy and postpartum in *ex vivo* assays. We did not conduct a power analysis prior to conducting this analysis as this was an exploratory pilot study and due to a lack of similar studies to reference to estimate effect sizes. Thus, we may be underpowered to detect small effect sizes. The Cronbach’s ɑ of the stress measure was low and results related to it should be interpreted with caution. This analysis was cross-sectional and thus not possible to investigate causal associations between maternal psychological distress and circulating Treg levels. Future longitudinal studies are needed to investigate pathways of causality.

## 5. Conclusion

In this study, we investigated the associations between maternal symptoms of depression, state anxiety, stress, and pregnancy-related anxiety in early and later pregnancy and peripheral levels of regulatory T-cell populations. We found that state anxiety symptoms and perceived stress were negatively associated with circulating Treg populations in early pregnancy. Only pregnancy-related anxiety was associated with Tregs in later pregnancy. We did not detect any statistically significant associations between symptoms of depression and Tregs at either early or later pregnancy. These results suggest that maternal symptoms of psychological distress may impact the tolerogenic function of the maternal immune system in pregnancy or that shifts in the immune system that occur during the perinatal period may affect the risk for the development of depression and anxiety symptoms in this period. It is crucial to elucidate the biological signature of psychological distress during pregnancy beyond constructs that happen to be routinely available in medical records or are inexpensive and easy to measure. In this regard, immunoregulation has been overlooked as a potentially critical mechanism to explain how and why prenatal psychological distress has been observed to be associated with deleterious downstream outcomes for mothers and offspring. Moreover, although Latinas experience high levels of symptoms of depression and anxiety during pregnancy, relatively little research is conducted with this population, and research is needed to understand biopsychosocial factors that contribute to the etiology of these disorders. Clarifying the mechanisms that confer risk for perinatal mood and anxiety disorders may also be useful for the development of possible biomarkers of clinical risk. Future studies are needed to examine the directionality of these associations, as well as the functional capacity of these Treg subpopulations. Longitudinal studies are also needed to track changes in Treg subpopulations and associations with mood and anxiety disorder symptoms across pregnancy and the postnatal periods. Such future studies can help clarify how these alterations shape maternal health risk and fetal and infant outcomes and identify possible opportunities for intervention.

## Supplementary Material

Supplement

## Figures and Tables

**Table 1:** Demographic and clinical characteristics for women participating in the study.

	All (n = 82)
**Age** (years)	
Mean (SD)	30.9 (5.68)
Median [Min, Max]	29.6 [21.4, 42.0]
**Country of birth** (%)	
US	37 (45.1 %)
Mexico	33 (40.2 %)
Others	12 (14.6 %)
**Education** (%)	
Less than high school	16 (19.5 %)
High school or equivalent	52 (63.4 %)
Any college or beyond	14 (17.1 %)
**Work or school status** (%)	
Employed	41 (50.0 %)
Unemployed	39 (47.6 %)
Missing	2 (2.4 %)
**Gestational age** (weeks)	
Mean (SD)	12.3 (2.89)
Median [Min, Max]	12.6 [5.14, 17.6]
**Number of children**	
Mean (SD)	1.41 (1.49)
Median [Min, Max]	1.00 [0, 6.00]
**First pregnancy** (%)	
No	63 (76.8 %)
Yes	19 (23.2 %)
**Marital status** (%)	
Single	42 (51.2 %)
Married	37 (45.1 %)
Missing	3 (3.7 %)
**Clinical depression status** (%)	
No (EPDS < 9)	63 (76.8 %)
Yes (EPDS ≥ 9)	19 (23.2 %)

EPDS = Edinburgh Postnatal Depression Scale.

## Data Availability

Data will be made available on request.
