# Supplementary material for: Regulatory T-cell phenotypes in prenatal psychological distress
Source: Brain Behav Immun. Author manuscript; Available in PMC 2024 Sep 15. (PMC11402516; doi:10.1016/j.bbi.2023.11.033)
Supplement: Supplement [file NIHMS2019670-supplement-Supplement.docx]

**Supplemental information**

**Table S1** Mental health measures. EPDS = Edinburgh Postnatal Depression Scale, PSS = Shortened Perceived Stress Scale, STAI = Spielberger State-Trait Anxiety Inventory, PRA = Pregnancy Related Anxiety Scale

|  | **n = 82** |
| --- | --- |
| **Gestational age** (weeks) |  |
| Mean (SD) | 12.3 (2.89) |
| Median [Min, Max] | 12.6 [5.14, 17.6] |
| **EPDS** |  |
| Mean (SD) | 5.10 (4.45) |
| Median [Min, Max] | 4.00 [0, 21.0] |
| **PSS** |  |
| Mean (SD) | 4.67 (2.71) |
| Median [Min, Max] | 4.50 [0, 9.00] |
| **STAI** |  |
| Mean (SD) | 10.4 (3.10) |
| Median [Min, Max] | 10.0 [6.00, 18.0] |
| **PRA** |  |
| Mean (SD) | 1.69 (0.560) |
| Median [Min, Max] | 1.60 [1.00, 3.20] |

**Table S2** Association of Tregs and mental health.

| Mental Health | Tregs | **Model1**: Gestational age | | | | **Model2**: Gestational age + First Pregnancy | | | | **Model3**: Gestational age + First Pregnancy + Number of Children | | | |
| --- | --- | --- | --- | --- | --- | --- | --- | --- | --- | --- | --- | --- | --- |
|  |  | b | LL | UL | p | b | LL | UL | p | b | LL | UL | p |
| EPDS | Foxp3 | -1.93 | -5.72 | 1.86 | 0.313 | -1.93 | -5.72 | 1.86 | 0.313 | -2.08 | -5.89 | 1.73 | 0.28 |
|  | Helios | -2.1 | -6.09 | 1.89 | 0.298 | -2.12 | -6.07 | 1.83 | 0.288 | -2.4 | -6.38 | 1.59 | 0.235 |
|  | CD45RA | -0.68 | -4.53 | 3.17 | 0.726 | -0.64 | -4.59 | 3.31 | 0.748 | -0.73 | -4.69 | 3.23 | 0.715 |
|  | CTLA4 | 0.2 | -5.43 | 5.83 | 0.944 | 0.16 | -5.44 | 5.77 | 0.954 | 0.14 | -5.48 | 5.76 | 0.961 |
|  | PD1 | -0.72 | -6.58 | 5.14 | 0.807 | -0.66 | -6.6 | 5.27 | 0.824 | -0.53 | -6.48 | 5.43 | 0.861 |
|  | TIGIT | -2.37 | -6.14 | 1.4 | 0.214 | -2.37 | -6.16 | 1.42 | 0.217 | -2.54 | -6.35 | 1.27 | 0.188 |
|  | TIM3 | -1.92 | -5.33 | 1.49 | 0.266 | -1.92 | -5.32 | 1.48 | 0.265 | -2.02 | -5.43 | 1.39 | 0.243 |
| PSS | Foxp3 | -1.5 | -3.8 | 0.79 | 0.196 | -1.13 | -3.35 | 1.1 | 0.317 | -1.33 | -3.5 | 0.85 | 0.23 |
|  | Helios | -0.21 | -2.66 | 2.23 | 0.862 | -0.16 | -2.5 | 2.17 | 0.892 | -0.5 | -2.8 | 1.81 | 0.67 |
|  | CD45RA | 0.61 | -1.73 | 2.95 | 0.603 | 1.36 | -0.94 | 3.66 | 0.242 | 1.24 | -1.02 | 3.5 | 0.278 |
|  | CTLA4 | -2.37 | -5.75 | 1.01 | 0.167 | -2.1 | -5.36 | 1.15 | 0.202 | -2.14 | -5.32 | 1.05 | 0.185 |
|  | **PD1** | **-4.02** | **-7.47** | **-0.57** | **0.023**** | -3.35 | -6.75 | 0.05 | 0.053 | -3.16 | -6.5 | 0.18 | 0.063 |
|  | TIGIT | -1.78 | -4.06 | 0.5 | 0.123 | -1.43 | -3.65 | 0.79 | 0.205 | -1.65 | -3.83 | 0.52 | 0.134 |
|  | TIM3 | -0.93 | -3.01 | 1.15 | 0.375 | -0.68 | -2.69 | 1.32 | 0.5 | -0.82 | -2.78 | 1.15 | 0.41 |
| STAI | **Foxp3** | **-2.88** | **-5.48** | **-0.27** | **0.031**** | **-2.8** | **-5.38** | **-0.23** | **0.033**** | **-2.86** | **-5.46** | **-0.25** | **0.032**** |
|  | **Helios** | **-3.29** | **-6.01** | **-0.56** | **0.019**** | **-3.24** | **-5.91** | **-0.57** | **0.018**** | **-3.36** | **-6.06** | **-0.65** | **0.016**** |
|  | CD45RA | -2.58 | -5.26 | 0.1 | 0.059 | -2.45 | -5.14 | 0.25 | 0.074 | -2.48 | -5.19 | 0.24 | 0.073 |
|  | CTLA4 | -0.58 | -4.6 | 3.45 | 0.777 | -0.57 | -4.47 | 3.33 | 0.771 | -0.58 | -4.5 | 3.35 | 0.77 |
|  | PD1 | -1.24 | -5.45 | 2.97 | 0.559 | -1.11 | -5.23 | 3.01 | 0.593 | -1.08 | -5.24 | 3.07 | 0.606 |
|  | TIGIT | -2.23 | -4.89 | 0.43 | 0.099 | -2.15 | -4.77 | 0.47 | 0.107 | -2.2 | -4.84 | 0.44 | 0.101 |
|  | **TIM3** | **-3.17** | **-5.5** | **-0.84** | **0.008**** | **-3.07** | **-5.36** | **-0.79** | **0.009**** | **-3.11** | **-5.41** | **-0.81** | **0.009**** |
| PRA | Foxp3 | -0.28 | -0.75 | 0.2 | 0.25 | -0.31 | -0.78 | 0.16 | 0.195 | -0.33 | -0.8 | 0.14 | 0.167 |
|  | Helios | -0.22 | -0.73 | 0.28 | 0.382 | -0.21 | -0.71 | 0.28 | 0.391 | -0.25 | -0.75 | 0.25 | 0.318 |
|  | CD45RA | -0.15 | -0.63 | 0.33 | 0.544 | -0.22 | -0.72 | 0.27 | 0.364 | -0.24 | -0.73 | 0.25 | 0.337 |
|  | CTLA4 | 0.34 | -0.36 | 1.04 | 0.334 | 0.32 | -0.37 | 1.02 | 0.361 | 0.32 | -0.38 | 1.01 | 0.366 |
|  | PD1 | 0.38 | -0.35 | 1.11 | 0.305 | 0.32 | -0.42 | 1.05 | 0.395 | 0.34 | -0.4 | 1.08 | 0.366 |
|  | TIGIT | -0.26 | -0.73 | 0.22 | 0.285 | -0.3 | -0.77 | 0.18 | 0.215 | -0.32 | -0.79 | 0.15 | 0.182 |
|  | TIM3 | -0.11 | -0.54 | 0.32 | 0.624 | -0.13 | -0.55 | 0.3 | 0.551 | -0.14 | -0.57 | 0.29 | 0.511 |

***p* < 0.05. EPDS = Edinburgh Postnatal Depression Scale, PSS = Shortened Perceived Stress Scale, STAI = Spielberger State-Trait Anxiety Inventory, PRA = Pregnancy-Related Anxiety Scale

**Figure S1. Definition of T-cell subsets and gating strategy.**

(**A**) Selection of live lymphocytes after gating for single and living cells. (**B**) Selection of CD3^+^ lymphocytes within the lymphocyte gate. (**C**) Selection of CD4^+^ and CD8^+^ lymphocytes within the CD3^+^ lymphocyte gate. (**D**) Selection of CD25^hi^CD127^lo^ cells within the CD4+ lymphocyte gate. (**E**) Selection of FoxP3+ T-regulatory (Tregs) cells within the CD25^hi^CD127^lo^ gate. (**F**) Selection of Helios^+^ and Helios^-^ cells within the FoxP3^+^ Tregs gate. **(G)** Selection of CD45RA+ cells within the FoxP3^+^ Tregs gate. **(H)** Selection of CTLA-4^+^ cells within the FoxP3^+^ Tregs gate. **(I)** Selection of PD-1^+^ cells within the FoxP3^+^ Tregs gate. **(J)** Selection of TIGIT^+^ cells within the FoxP3^+^ Tregs gate. **(K)** Selection of TIM-3^+^ cells within the Foxp3^+^ Tregs gate.


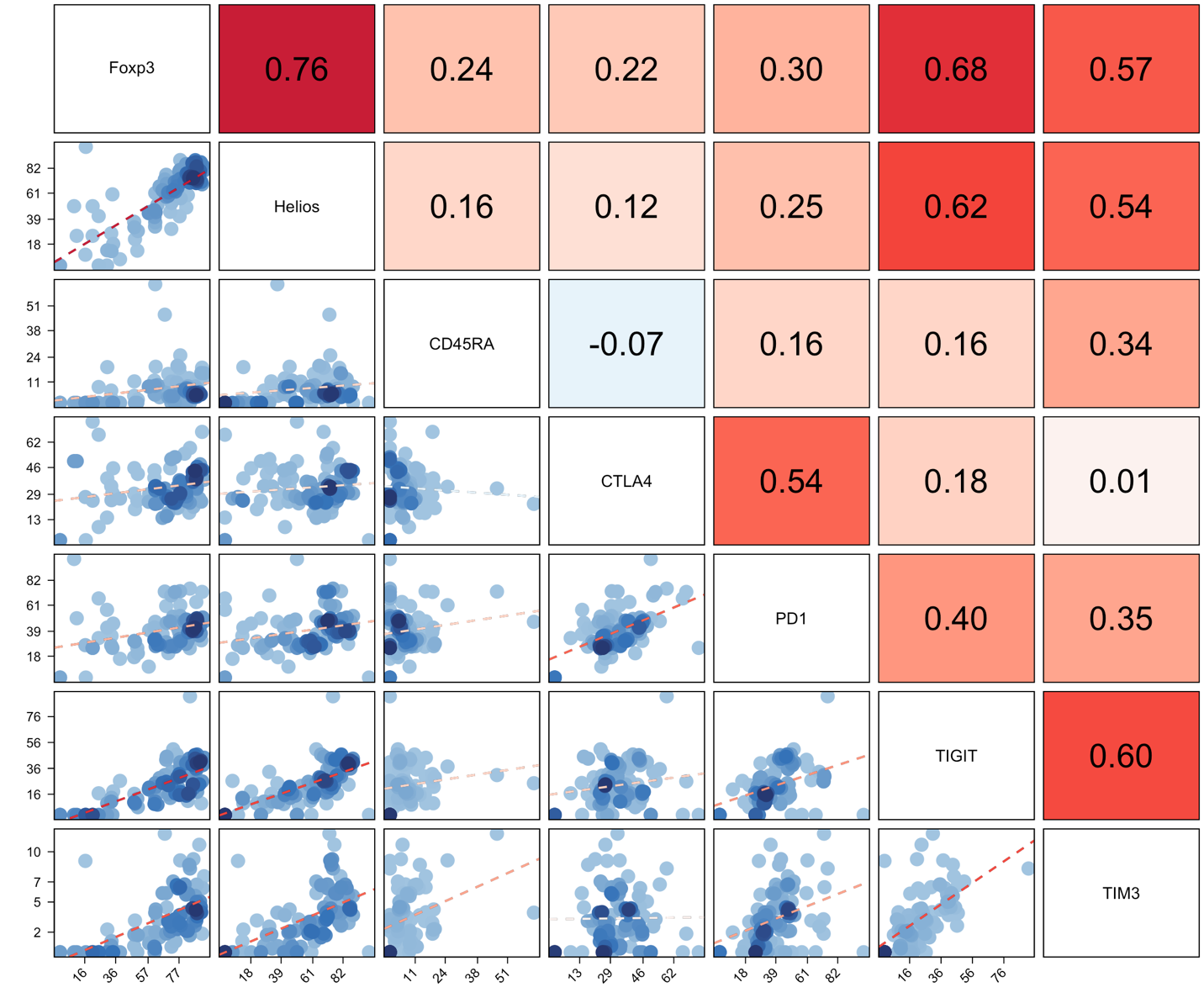


**Figure S2** Correlations among 7 Treg populations in pregnant women. The figure shows a matrix of correlations illustrating relationships among 7 Treg populations: Foxp3, Helios, CD45RA, CTLA-4, PD-1, TIGIT, TIM-3. Data are for n = 82 women with complete data on all Tregs. The diagonal cells of the matrix list the Tregs. The half of the matrix below the diagonal shows scatter plots of associations. For each scatter-plot cell, the y-axis corresponds to the variable name along the matrix diagonal to the right of the plot and the x-axis corresponds to the variable named along the matrix diagonal above the plot. The half of the matrix above the diagonal list Pearson correlations between the Tregs. For each correlation cell, the value reflects the correlation of the variables named along the matrix diagonal to the left of the cell and below the cell.
